# Prädiktive Immunzytochemie beim nicht-kleinzelligen Lungenkarzinom

**DOI:** 10.1007/s00292-022-01066-4

**Published:** 2022-04-11

**Authors:** Luka Brcic, Spasenija Savic Prince

**Affiliations:** 1 Diagnostik und Forschungsinstitut für Pathologie, Medizinische Universität Graz, Graz, Österreich; 2 Pathologie, Institut für Medizinische Genetik und Pathologie, Universitätsspital Basel, Schönbeinstrasse 40, 4031 Basel, Schweiz

**Keywords:** Prädiktiv, Immunzytochemie, Lungenkarzinom, Zytologie, Biomarker, Predictive, Immunocytochemistry, Lung cancer, Cytology, Biomarkers

## Abstract

Die Immunchemie ist eine zeit-, tumorproben- und kosteneffiziente Methode zur Untersuchung prädiktiver Biomarker bei fortgeschrittenen nicht-kleinzelligen Lungenkarzinomen (NSCLC). Die Immunhistochemie (IHC) an Formalin-fixiertem, Paraffin-eingebettetem (FFPE) Tumorgewebe hat sich für den Nachweis der PD-L1-Expression sowie für die ALK-, ROS1- und neuerdings auch für die NTRK-Untersuchung bewährt. Zytologische Proben als Quelle für prädiktive Markeranalysen sind sehr wichtig, da bis zu 40 % aller NSCLC rein zytologisch diagnostiziert werden.

Trotz der etablierten Rolle der Zytologie in der Lungenkarzinomdiagnostik wurden keine kommerziellen IHC-Assays für zytologische Proben validiert.

Die prädiktive Immunzytochemie (ICC) ist am einfachsten an FFPE-Zellblöcken (CB) durchzuführen, da für FFPE-Histologie standardisierte Protokolle verwendet werden können. CB sind jedoch nicht immer verfügbar.

Nicht als CB verarbeitete zytologische Präparate sind weniger standardisiert als histologische Präparate und weisen eine erhebliche präanalytische Variabilität auf. Daher ist eine strenge zytologiespezifische Optimierung, Validierung und Qualitätskontrolle von ICC-Protokollen erforderlich. Unter dieser Voraussetzung ist die prädiktive ICC, die in der Regel an Papanicolaou-gefärbten Zytologien durchgeführt wird, robust und zuverlässig. Dieses wertvolle zytologische Material sollte für prädiktive Biomarkeranalysen genutzt werden, um Patientinnen und Patienten nicht dem unnötigen Risiko einer erneuten Probenentnahme auszusetzen. Diese Übersichtsarbeit beleuchtet präanalytische, analytische und postanalytische Aspekte, die ICC-Ergebnisse beeinflussen können, und fasst die veröffentlichten Daten zur prädiktiven ICC für PD-L1, ALK und ROS1 bei NSCLC zusammen.

## Abstract (en)

Predictive immunochemistry is a time-, tumor sample- and cost-efficient method for testing the increasing number of predictive biomarkers in advanced non-small cell lung cancer (NSCLC). Immunohistochemistry (IHC) on formalin-fixed, paraffin-embedded (FFPE) tumor tissue has an established role in detecting PD-L1 expression and in ALK, ROS1, and more recently NTRK testing. Cytology specimens as a source for predictive biomarker testing in NSCLC is very important as up to 40% of all NSCLC are diagnosed by cytology alone.

Despite the established role of cytology in lung cancer diagnosis, no commercial IHC assays have been validated for cytology specimens.

FFPE cell blocks (CB) are the most straightforward cytology preparation for predictive immunocytochemistry (ICC) as the results are valid using protocols standardized for FFPE histology. But CB are not always available.

With non-CB cytology specimens being less standardized than FFPE histology and with considerable preanalytical variability, rigorous cytology-specific ICC protocol optimization, validation, and quality control are required. With this prerequisite, predictive ICC, most commonly performed on Papanicolaou-stained cytology specimens, is robust and reliable on non-CB preparations. This valuable material should not be underutilized for predictive biomarker testing, as this would put patients at risk of unnecessary repeat sampling. This review highlights preanalytical, analytical, and postanalytical aspects that may influence ICC results and summarizes the published data on predictive ICC for PD-L1, ALK, and ROS1 in NSCLC.

Die Immunhistochemie (IHC) zur Bestimmung von prädiktiven Biomarkern ist beim nicht-kleinzelligen Lungenkarzinom (NSCLC) gut etabliert. Sie liefert zeitnah therapeutisch relevante Ergebnisse und ist für die Selektion von Patientinnen und Patienten für eine Erstlinien-Immuntherapie unerlässlich. Bei bis zu 40 % aller Lungenkarzinome sind zytologische Proben das einzige Tumormaterial, welches für prädiktive Biomarkeranalysen zur Verfügung steht. Eine zuverlässige prädiktive Immunzytochemie (ICC) ist daher ein klinisch relevanter Bedarf.

Die Behandlungsmöglichkeiten für Patientinnen und Patienten mit einem fortgeschrittenen, inoperablen nicht-kleinzelligen Lungenkarzinom (NSCLC), haben sich erheblich verbessert. In der Regel werden NSCLC durch kleine Biopsien und/oder zytologische Proben diagnostiziert. Prädiktive Biomarker einschließlich des morphologischen Subtyps, des PD-L1-Status und der Ergebnisse von zielgerichteten onkogenen Treiberveränderungen stehen in direktem Zusammenhang mit der Wahl einer spezifischen onkologischen Therapie [7].

Angesichts der wachsenden Zahl behandlungsrelevanter Biomarker kann die prädiktive Immunchemie zeit-, tumorproben- und kosteneffiziente Ergebnisse liefern. Sie ist bei Formalin-fixiertem, Paraffin-eingebettetem (FFPE) Tumorgewebe für die PD-L1-, ALK-, ROS1- und neuerdings auch für die NTRK-Testung gut etabliert [12, 18, 24, 27].

Im Allgemeinen ist die ICC an zytologischen Proben gängige Praxis und liefert Ergebnisse von guter Qualität. Eine Herausforderung für robuste und zuverlässige ICC-Ergebnisse besteht darin, dass die präanalytische Verarbeitung von zytologischen Proben im Vergleich zu histologischen Proben weit weniger standardisiert ist und eine zytologiespezifische Protokolloptimierung, Validierung und Qualitätskontrolle erfordert [8]. Diese Übersichtsarbeit beleuchtet präanalytische, analytische und postanalytische Aspekte, die die ICC-Ergebnisse beeinflussen können, und fasst die veröffentlichten Daten der prädiktiven ICC für PD-L1, ALK und ROS1 bei NSCLC zusammen.

## Immunzytochemie

### Allgemeine präanalytische, analytische und postanalytische Aspekte

Zytologische Proben können auf unterschiedliche Weise verarbeitet werden: als konventionelle Abstriche, Zytospinpräparate oder Flüssigzytologien sowie FFPE-Zellblöcke (CB) [8]. Der Einfachheit halber werden alle nicht als CB verarbeiteten zytologischen Proben in diesem Artikel als konventionelle Zytologie bezeichnet.

Ein FFPE-CB ist die einfachste zytologische Präparationsart für die ICC, da standardisierte Protokolle, welche für histologische Proben etabliert wurden, verwendet werden können. Im Allgemeinen sind die Ergebnisse der ICC an CB robust und reproduzierbar. Es sollte jedoch bedacht werden, dass es keine standardisierte Methode für die CB-Herstellung gibt. So werden z. B. unterschiedliche Entnahmemedien und Vorfixierungsmittel (Ethanol, Formalin, Methanol) verwendet, die die Immunreaktivität beeinflussen und Anpassungen des IHC-Protokolls erfordern können [8, 9].

CB sind aber nicht immer verfügbar und weisen häufig eine zu tiefe Zellularität auf. Eine kürzlich durchgeführte Umfrage unter europäischen Labors hat gezeigt, dass Papanicolaou(Pap)-gefärbte oder luftgetrocknete konventionelle Zytologien immer noch das wichtigste zytologische Material für die ICC sind [25]. Eine ICC auf dem diagnostischen, Pap-gefärbten Zytologiepräparat stellt sicher, dass die zu untersuchenden Zielzellen vorhanden sind. Es wird empfohlen, in der Lungenzytologie positiv geladene Objektträger zu verwenden, da dies die Adhärenz der Zellen verbessert und verhindert, dass sie sich während der technischen ICC-Verarbeitung ablösen und verloren gehen. Die Pap-Färbung beeinträchtigt die ICC nicht und es ist kein separater Entfärbungsschritt erforderlich [8].

Konventionelle Zytologien sind Präparate mit hoher präanalytischer Variabilität (unterschiedliche Entnahmeverfahren, Konservierungs- und Transportmedien, Präparationsverfahren und Fixationslösungen). Daher sind in der Regel zytologiespezifische Anpassungen der Analysevariablen und eine separate Validierung der ICC-Protokolle erforderlich, da sich die präanalytische Aufbereitung deutlich von FFPE-Proben unterscheidet.

Zahlreiche analytische Variablen können die Färbereaktion der Immunchemie beeinflussen, darunter die Sensitivität und Spezifität des primären Antikörpers, die Antikörperkonzentration, die Bedingungen für die Antigendemaskierung, die Sensitivität der Nachweismethode und die Kalibrierung der Färbung mit geeigneten Positivkontrollen. Im Vergleich zu IHC-Protokollen erfordern ICC-Protokolle für konventionelle zytologische Proben oft keine oder eine geringere Vorbehandlung, und oft muss die Antikörperverdünnung angepasst werden [8].

Postanalytisch ist die Identifikation der zu untersuchenden Karzinomzellen auf dem Objektträger und die Anwendung adäquater Auswertekriterien entscheidend für eine zuverlässige ICC-Interpretation. Nicht-neoplastische Zellen, insbesondere Makrophagen, können mit verschiedenen Antikörpern reagieren. Dreidimensionale Zellverbände können eine unspezifische Immunfärbung im Zentrum der Verbände aufweisen [19]. Degenerierte Zellen und Nekrosen können ebenfalls zu unspezifischen Färbereaktionen führen und sollten bei der Beurteilung nicht berücksichtigt werden [8].

Erwähnenswert ist, dass die ICC die DNA nicht schädigt, sodass ICC-gefärbte Zytologiepräparate für weitere molekulare prädiktive Analysen verwendet werden können. Die Fluoreszenz-in-situ-Hybridisierung (FISH) ist an ICC-gefärbten Proben gut anwendbar, wenn 3‑Amino-9-Ethylcarbazol (AEC) als Chromogen verwendet wird, während 3,3-Diaminobenzidin (DAB) die FISH-Signale aufgrund einer Autofluoreszenz stark überlagert [20].

### Analytische Validierung und Qualitätskontrolle

Zur Erstellung und Optimierung eines ICC-Protokolls sind Positiv- und Negativkontrollen erforderlich. Die Kontrollproben müssen auf die gleiche Weise verarbeitet und fixiert werden wie die klinischen Proben. Positive und negative Zytologiekontrollen können aus handelsüblichen Zellkulturen (erhältlich für ALK, ROS1 und PD-L1), aus übrig gebliebenen Ergussflüssigkeiten oder aus Frischgewebeabstrichen von Resektionspräparaten (Lungenkarzinomresektate, für PD-L1 auch Plazenta) hergestellt werden [8]. Eine weitere Validierung sollte an einer Reihe von klinischen NSCLC-Proben erfolgen, indem die ICC-Ergebnisse mit dem Goldstandard verglichen werden. Dabei kann es sich um gepaarte histologische Proben mit validierten IHC-Ergebnissen oder mit Ergebnissen molekularer Analysen (z. B. für ALK, ROS1 oder pan-TRK) handeln. Im Allgemeinen gilt ein laborentwickelter Test (LDT) als technisch valide, wenn er mindestens 90 % Übereinstimmung mit dem Referenztest aufweist.

Zytologiespezifische Empfehlungen für die analytische Validierung von prädiktiven ICC-Protokollen sind nicht verfügbar; es wurden jedoch allgemeine Empfehlungen für die analytische Validierung vorgeschlagen, um genaue und reproduzierbare immunchemische prädiktive Resultate zu gewährleisten [3]. Zu diesen Empfehlungen gehört, dass für die anfängliche analytische Validierung eines neuen prädiktiven Protokolls mindestens 20 positive und 20 negative Kontrollen getestet werden sollten, was für ALK, ROS1 und pan-TRK unrealistisch ist, da die Prävalenz dieser onkogenen Fusionen bei NSCLC sehr tief ist.

Die Verwendung eines ICC-Protokolls, welches bereits von einem anderen Labor validiert wurde, kann die Einführung eines neuen ICC-Tests vereinfachen. Da die präanalytischen Bedingungen weniger standardisiert sind als bei FFPE-Proben und die lokalen Bedingungen variieren können, ist eine Validierung erforderlich, um das Protokoll bei Bedarf auf die lokalen Bedingungen anzupassen.

Nach Einführung eines neuen prädiktiven ICC-Tests in die klinische Diagnostik, bietet ein prospektives Monitoring der ICC-Ergebnisse (z. B. Prävalenz der PD-L1-Expressionsniveaus bei verschiedenen Grenzwerten und Prävalenz von ALK-, ROS1- und pan-TRK-positiven Ergebnissen) eine kontinuierliche Qualitätskontrolle und ist hilfreich, um Veränderungen in der analytischen Testleistung zu erkennen und um reproduzierbare Resultate sicherzustellen [3]. So wird die Bioplaza-Onlineplattform beispielsweise von mehreren Pathologielabors in Europa genutzt, um ihre kodierten PD-L1-Ergebnisse prospektiv zu verfolgen und die Prävalenz positiver Ergebnisse mit dem jeweiligen nationalen Durchschnitt zu vergleichen [6].

Die externe Qualitätskontrolle (EQC) ist ein wichtiges Instrument zur Qualitätssicherung. Derzeit gibt es nur einen EQC-Dienst (das UK NEQAS-Zytologiemodul) für die ICC an zytologischen Proben. Bisher bietet es nur Module für eine begrenzte Anzahl von ICC-Markern, wobei PD-L1, ALK oder ROS1 noch nicht beinhaltet sind. Dies unterstreicht die Notwendigkeit einer internen Qualitätskontrolle und einer Ausweitung der auf zytologische Proben zugeschnittenen EQC-Angebote [9, 25].

## PD-L1

Eine Untersuchung des PD-L1-Status ist bei metastasierten NSCLC für alle histologischen Subtypen erforderlich, um Patientinnen und Patienten für eine Pembrolizumab-Monotherapie zu identifizieren [18]. Bei metastasierten NSCLC mit hoher PD-L1-Expression (Tumor Proportion Score, TPS, ≥ 50 %) und negativem Nachweis zielgerichtet angehbarer onkogener Treiberalterationen ist die Pembrolizumab-Monotherapie die Erstlinienbehandlung der Wahl.

Die PD-L1-Untersuchung kann an FFPE-Tumorgewebe mit hoch standardisierten, in klinischen Studien validierten, PD-L1-IHC-Assays durchgeführt werden oder mit weniger kostspieligen PD-L1-IHC-LDT, die nicht an einen spezifischen Färbeautomaten gebunden sind [11].

Keiner der kommerziellen PD-L1-Assays wurde von den Herstellern für zytologische Proben validiert.

Studienergebnisse zeigen, dass für FFPE-CB Histologie-standardisierte PD-L1-IHC-Protokolle gut verwendet werden können. Mehrere Studien zeigen übereinstimmende PD-L1-Ergebnisse mit gepaarten histologischen NSCLC-Proben sowohl für kommerzielle PD-L1-Assays als auch für LDT. Die Gesamtkonkordanzrate liegt dabei > 90 % bei Verwendung des klinisch relevanten TPS-Grenzwertes von 50 % [4].

Publizierte Daten für konventionelle Zytologien sind noch begrenzt. Erste retrospektive Studien haben an Pap-gefärbten zytologischen Präparaten eine hohe Übereinstimmung der PD-L1-Ergebnisse mit gepaarten FFPE-Proben gezeigt, sowohl bei Verwendung des 22C3- als auch des SP263-Antikörperklons [13, 17]. Die Daten sind jedoch uneinheitlich, da nachfolgende Studien unterschiedliche Konkordanzraten aufwiesen, was wahrscheinlich auf eine unzureichende Validierung zurückzuführen ist [2, 16].

Für die Etablierung eines neuen PD-L1-ICC-Protokolles sind als Positivkontrollen Pap-gefärbte Zytospins der Karpas-299- (diffuse PD-L1-Färbung mit mittlerer Intensität) und der LNCap-Zelllinien (fokale PD-L1-Färbung mit schwacher Intensität) gut geeignet. Zur Validierung der PD-L1-ICC sollten die Positivkontrollen im klinischen Validierungsset (z. B. Pap-gefärbte NSCLC-Zytologieproben mit bekanntem PD-L1-IHC-Status gepaarter Histologien) das gesamte Spektrum der PD-L1-Färbeintensitäten bei unterschiedlichen Expressionsniveaus (einschließlich schwacher Färbung und geringem Anteil gefärbter Zellen) repräsentieren, um eine angemessene Kalibrierung der ICC zu gewährleisten.

In der diagnostischen Routine am Universitätsspital Basel werden 35 % aller PD-L1-Untersuchungen bei NSCLC an Pap-gefärbten zytologischen Präparaten durchgeführt (berechnet aus > 1000 PD-L1-Ergebnissen, Daten nicht gezeigt). Die PD-L1-ICC entspricht einem SP263 LDT auf der BOND-MAX-Färbemaschine (Leica Biosystems GmbH, Deutschland). Als kontinuierliche QC-Maßnahme wird die Prävalenz der PD-L1-Resultate bei verschiedenen Expressionsgrenzwerten (TPS < 1 %, 1–49 % bzw. 50 %) prospektiv mit der Bioplaza-Onlineplattform überwacht. Die Prävalenz von NSCLC mit hoher PD-L1-Expression (TPS ≥ 50 %) ist dabei vergleichbar zwischen Biopsien (unter Verwendung des SP263-Assays) und Pap-gefärbten zytologischen Präparaten (30 % bzw. 27 %, *p* = 0,49) und entspricht den erwarteten Werten der publizierten Literatur. Diese Daten aus der klinischen Routinepraxis unterstreichen, dass die PD-L1-ICC an konventionellen Pap-gefärbten Präparaten zuverlässige Resultate für den klinisch relevanten Grenzwert (TPS ≥ 50 %) liefern kann.

Eine kürzlich veröffentlichte nationale Studie aus den Niederlanden, bei der retrospektiv Daten aus der diagnostischen Routine ausgewertet wurden, zeigte jedoch eine größere Variabilität der PD-L1-Positivitätsraten zwischen verschiedenen Labors bei zytologischen Proben im Vergleich zu histologischen Proben (bei einem TPS-Grenzwert von 50 %). Dies untermauert die Notwendigkeit einer sorgfältigen Etablierung und Validierung von PD-L1-ICC-Protokollen sowie von Qualitätskontrollmaßnahmen [10].

### PD-L1-Immunzytochemie: Auswertekriterien

Für eine zuverlässige Beurteilung des PD-L1-Status müssen mindestens 100 vitale Tumorzellen vorhanden sein[8]. An CB gelten für Tumorzellen die gleichen Auswertekriterien wie für FFPE-Histologien. Eine Tumorzelle ist positiv für PD-L1, wenn eine teilweise oder vollständige Membranfärbung vorhanden ist, unabhängig von der Intensität der Färbung. Nekrotische Tumorzellen und eine zytoplasmatische Färbereaktion werden nicht berücksichtigt. Bei CB kann es schwierig sein, Tumorzellen von umliegenden nicht-neoplastischen Zellen, z. B. von Makrophagen, zu unterscheiden, was zu Fehlinterpretationen führen kann. Eine Immunfärbung mit einem Epithelmarker (z. B. TTF‑1, BerEp4) auf einem entsprechenden Schnitt kann bei der Identifizierung von Tumorzellen für die Bewertung der PD-L1-Expression nützlich sein. Makrophagen weisen zudem häufig eine membranäre PD-L1-Expression auf und können als interne Positivkontrolle dienen.

**Figure 1:**
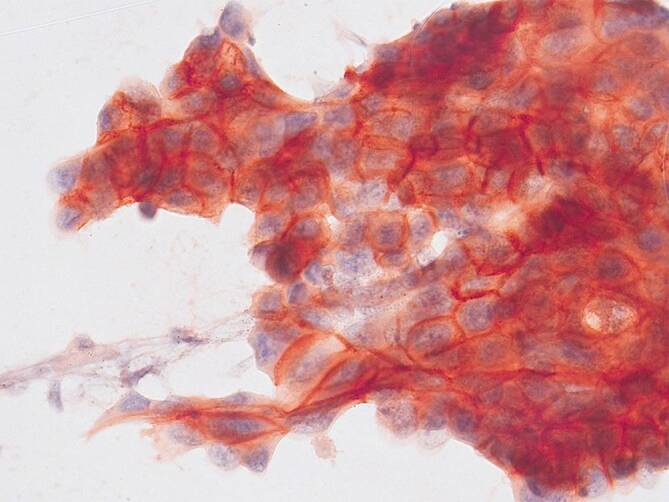

Bei konventionellen Zytologien sind die Zellen auf den Objektträgern nicht angeschnitten und weisen daher eine intakte Zellmembran auf. Durch diese intakte Zellmembran ist die membranäre Immunfärbung weniger deutlich erkennbar und erscheint als diffuses „pseudozytoplasmatisches“ PD-L1-Färbemuster [8, 26]. In vielen Fällen ist jedoch eine membranäre Akzentuierung der Immunfärbung zu erkennen (Abb. 1).

Die PD-L1-Expression ist nicht auf Tumorzellen beschränkt, sondern wird auch von Immunzellen (IC) exprimiert. Eine PD-L1-Auswertung von tumorassoziierten IC ist an zytologischen Präparaten nicht zuverlässig möglich, da eine Unterscheidung zwischen tumorassoziierten IC und IC außerhalb des Tumorbettes nicht möglich ist. Beim NSCLC ist der PD-L1-Status jedoch nur für die Verordnung eines Medikaments, nämlich Pembrolizumab, verpflichtend notwendig und für den dafür benötigte TPS werden nur Tumorzellen in die Auswertung einbezogen.

## Prädiktive Immunzytochemie als Surrogat für onkogene Rearrangements: ALK, ROS1 und pan-TRK

Bei metastasierten NSCLC sind zielgerichtete Medikamente für *ALK*-, *ROS1*-, *NTRK*- und neuerdings auch *RET*-Fusionen zugelassen. Insgesamt handelt es sich um seltene Alterationen mit einer Prävalenz von *ALK*-, *ROS1*-, *RET*- und *NTRK*-Rearrangements von 3–5 %, 1–2 %, 1–2 % bzw. 0,2 % in kaukasischen Populationen [24].

Bei onkogenen Genfusionen handelt es sich um Tyrosinkinasen, die aus strukturellen Umlagerungen auf DNA-Ebene resultieren. Wichtig ist, dass die Fusion die Funktion der Tyrosinkinase erhält und für die Überexpression des Fusionsproteins und die konstitutive Aktivierung der Kinase optimiert ist. Die immunchemisch nachgewiesene ALK-, ROS1- und pan-TRK-Expression ist ein Surrogat für die jeweiligen Rearrangements, da die Proteine dieser Gene in ihrer nativen Form im Wesentlichen nicht exprimiert werden.

Obwohl sich angesichts der steigenden Anzahl therapierelevanter genetischer Alterationen bei NSCLC ein Upfront DNA- und RNA-basiertes Next Generation Sequencing (NGS) anbietet mit gleichzeitiger Untersuchung prädiktiver Mutationen, Amplifikationen und Fusionen, ist ein substanzieller Anteil kleiner NSCLC-Proben nicht für eine zusätzliche RNA-basierte NGS-Analyse ausreichend [24]. Der DNA-basierte Nachweis von Rearrangements mittels Break-apart-FISH erfordert nur wenige Tumorzellen (meist nur 50), ist jedoch eine kostspielige Screeningmethode und erfordert ein hohes Maß an Fachwissen. Der immunchemische Nachweis von Fusionen auf Proteinebene ist eine breit verfügbare, kosteneffiziente Screeningmethode mit schneller Durchlaufzeit und kann einfach in prädiktive Testalgorithmen implementiert werden [15].

Im Gegensatz zur PD-L1-Immunchemie, bei der die PD-L1-Expression eine kontinuierliche Variabel mit beträchtlicher Heterogenität darstellt, liefert die Immunchemie bei onkogenen Fusionen in der Regel eine schwarz-weißes Resultat. Die meisten NSCLC mit einem *ALK*-, *ROS1*- oder *NTRK*-Rearrangement weisen ein deutlich sichtbares, diffuses immunchemisches Färbemuster der entsprechenden Proteine auf, was die Auswertung einfach macht. Da die Immunfärbung diffus und homogen über die Tumorzellen verteilt ist, reichen Proben mit nur 20 Tumorzellen für die Auswertung sowohl an histologischen als auch an zytologischen Proben aus [8].

### ALK

Auf Grundlage vergleichender Studienergebnisse ist die ALK-IHC unter Verwendung des 5A4- (LDT) oder D5F3-Antikörpers (LDT oder Assay) eine gleichwertige Alternative zur *ALK*-FISH, die früher als Goldstandard für die ALK-Untersuchung galt. Ein positives ALK-IHC-Ergebnis ist ausreichend für eine Behandlung mit einem ALK-Tyrosinkinase-Inhibitor [12]. Der automatisierte D5F3-ALK-Assay für BenchMark-Färbegeräte (Ventana Medical Systems, Inc., USA) kann die Einführung der ALK-IHC für FFPE-Proben erleichtern, da er keine umfangreiche lokale Revalidierung erfordert.

Für FFPE-CB zeigen ALK-IHC-Protokolle mit 5A4 oder D5F3 eine gute Übereinstimmung der Resultate mit der *ALK*-FISH. Mehrere Studien zeigen im Vergleich zur FISH eine Sensitivität von 100 %, wobei die Spezifität etwas variabler ist und zwischen 83 und 100 % liegt [8].

Die ALK-ICC kann an konventionellen zytologischen Proben eine hohe Übereinstimmung mit *ALK*-FISH- oder ALK-IHC-Ergebnissen von gepaarten histologischen NSCLC-Proben erzielen. Die Spezifitäten liegen zwischen 97 und 100 %. Die Sensitivitäten sind jedoch variabler (66–100 %), was die Notwendigkeit einer strengen Validierung und Qualitätskontrolle unterstreicht [19]. Es sollte auch bedacht werden, dass FISH ein schwieriger Goldstandard ist mit einer Rate an berichteten falsch positiven Resultaten von > 10 %, und das in erfahrenen FISH Labors [19, 21, 23].

**Figure 2:**
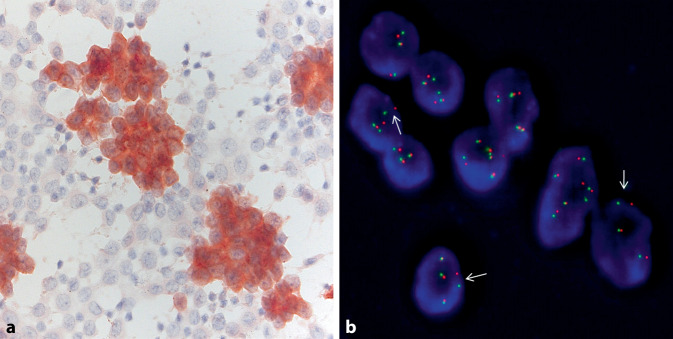

Bei ALK-positiven NSCLC ist die ALK-Immunfärbung in der Regel zytoplasmatisch und diffus in allen Tumorzellen vorhanden und zeigt eine mittlerer bis starke Intensität (Abb. 2). Im Gegensatz zur ALK-IHC an histologischen Proben sollte ein positives ALK-ICC-Ergebnis durch eine molekulare Methode bestätigt werden. Die Schwelle für die Einleitung einer molekularen Analyse sollte niedrig sein, um eine hohe Sensitivität zu gewährleisten. Selbst weniger als 20 positive Tumorzellen, unabhängig von der Färbeintensität, sollten als diagnostisch angesehen werden und eine Bestätigungsuntersuchung mittels FISH oder NGS auslösen.

### ROS1

Für ROS1 gibt es 2 verfügbare Antikörperklone, den D4D6 (Cell Signaling Technology, USA) und den später eingeführten SP384 (Ventana Medical Systems, Inc.). Im Gegensatz zu ALK gibt es für ROS1 keinen kommerziellen IHC-Assay.

Die D4D6-ROS1-IHC ist hochsensitiv für den Nachweis von *ROS1*-Rearrangements. Da die Spezifität im Vergleich zur ALK-IHC variabler ist, muss ein positives ROS1-IHC-Ergebnis durch eine molekulare Methode bestätigt werden, bevor eine Behandlung eingeleitet werden kann [12].

**Figure 3:**
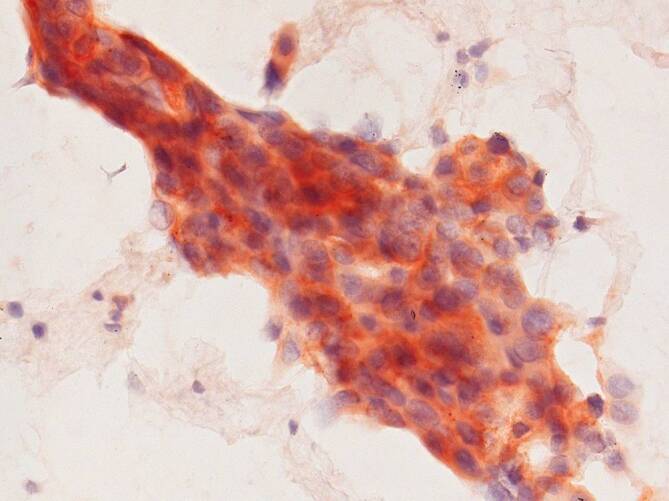

FFPE-CB und Pap-gefärbte Zytologiepräparate sind für die ROS1-ICC geeignet [1, 22, 28]. Vlajnic et al. wiesen an 295 prospektiven Pap-gefärbten zytologischen NSCLC-Proben identische Ergebnisse für die D4D6-ROS1-ICC und für molekulare Untersuchungen (*ROS1*-FISH oder RNA-basierter NGS) nach. Die ROS1-ICC detektierte 13 ROS1-positive NSCLC (Sensitivität und Spezifität von 100 %) [28]. Die Merkmale der ROS1-ICC-Färbung sind mit der ROS1-IHC an histologischen Proben vergleichbar. Positive NSCLC zeigen in der Regel eine diffuse zytoplasmatische Färbung, obwohl die Färbung heterogen sein und die Intensität der Färbung zwischen den Tumorzellen variieren kann (Abb. 3; [1]). Wie bei ALK sollte auch hier der Schwellenwert für die Einleitung einer molekularen Analyse niedrig gehalten werden.

Unspezifische ROS1 Färbungen können bei nicht-neoplastischen Zellen, insbesondere bei reaktiven Typ-II-Pneumozyten und Makrophagen vorkommen [1].

### NTRK

*NTRK* umfasst 3 Gene, *NTRK1*, -*2* und ‑*3*, die für die Transmembran-Rezeptor-Tyrosinkinasen TRKA, -B bzw. -C kodieren. Die Häufigkeit von *NTRK*-Fusionen ist bei NSCLC sehr gering (0,02 %) und kann alle 3 *NTRK*-Gene betreffen [24]. Bei einer so tiefen Prävalenz ist die NTRK-FISH kein geeignetes Instrument für das Screening bei NSCLC, da für jedes der 3 *NTRK*-Gene 3 separate FISH-Tests erforderlich sind.

TRKA, -B und -C weisen einen hohen Grad an Homologie zwischen den Kinasedomänen auf und können alle mit dem pan-TRK-Antikörperklon EPR17341 (Abcam, Cambridge, UK) nachgewiesen werden. Ein kommerzieller CE-IVD pan-TRK(EPR17341)-Assay für BenchMark-Färbeautomaten (Ventana Medical Systems, Inc.) ist für FFPE-Proben erhältlich.

Es gibt noch keine veröffentlichten zytologiespezifischen Daten zur pan-TRK-ICC.

Die Expression von TRKA, -B und -C in adulten Geweben ist auf neurale Komponenten (z. B. kortikales Gehirn) und die Hoden beschränkt. Diese Gewebe können zur Erstellung von Positivkontrollen für die Etablierung einer pan-TRK-ICC verwendet werden. Die TRK-Expression kann in Intensität und subzellulärer Lokalisierung variieren, ist aber in den meisten Fällen zytoplasmatisch. Eine nukleäre Expression wird typischerweise bei *NTRK3*-Fusionen beobachtet [5]. Eine TRK-Färbung in ≥ 1 % der Tumorzellen gilt als positives IHC-Ergebnis, da Tumore mit einer *NTRK3*-Fusion eine sehr fokale oder schwache TRK-Färbung aufweisen können. Die Sensitivität der pan-TRK-IHC ist hoch für *NTRK1* und *NTRK2* (96,2 bzw. 100 %), aber nur 79,4 % für *NTRK3*-Fusionen. *NTRK3*-Fusionen können somit verpasst werden, sind aber äußerst selten bei NSCLC. Die Gesamtsensitivität und -spezifität der ICC beträgt bei NSCLC 87,5 % bzw. 100 % [24]. Nach den aktuellen Empfehlungen muss ein positives pan-TRK-IHC-Ergebnis noch durch eine molekulare Methode (FISH oder RNA-basiertes NGS) bestätigt werden [14].

## Fazit für die Praxis

- Zytologische Proben sollten für prädiktive Biomarkeranalysen genutzt werden, um Patientinnen und Patienten nicht dem unnötigen Risiko einer erneuten Probenentnahme auszusetzen.
- Immunzytochemische (ICC) Untersuchungen an zytologischen Proben sind gängige Praxis und für die Diagnose und prädiktive Biomarkeranalysen unverzichtbar geworden.
- An FFPE-Zellblöcken (formalinfixiert und paraffineingebettet, FFPE) kann die prädiktive ICC in der Regel zuverlässig mit standardisierten Protokollen, die für histologische Proben entwickelt wurden, durchgeführt werden.
- Da sich konventionelle zytologische Proben deutlich von FFPE-Proben unterscheiden, erfordert die Etablierung von prädiktiven ICC-Protokollen meist eine zytologiespezifische Anpassung der analytischen Variablen und eine separate Validierung.
- Qualitätskontrollmaßnahmen sind von entscheidender Bedeutung, um eine hohe Qualität prädiktiver ICC-Ergebnisse zu gewährleisten.
